# Enhanced Recovery After Cardiac Transplantation: From Concept to Clinical Pathway

**DOI:** 10.21470/1678-9741-2026-0003

**Published:** 2026-09-08

**Authors:** Gabrielle Barbosa Borgomoni, Ligia Neres Matos, Diego Pereira Gregório de Andrade, Tauanny Stephane Frazão e Silva, Juan Cosquillo Mejía, Maurilio Onofre Deininger, Luiz Fernando dos Reis Falcão, Fernando Augusto Figueira, Omar Asdrúbal Vilca Mejia

**Affiliations:** 1 Department of Thoracic and Cardiovascular Surgery, Unidade Cirúrgica de Qualidade e Segurança, Instituto do Coração, Hospital das Clínicas da Faculdade de Medicina da Universidade de São Paulo (HCFMUSP), São Paulo, São Paulo, Brazil; 2 Cardiothoracic Transplantation Center, Hospital Copa Star – Rede D’or, Rio de Janeiro, Rio de Janeiro, Brazil; 3 Department of Heart Transplantation, Hospital do Coração Alagoano Prof Adib Jatene, Maceió, Alagoas, Brazil; 4 Programa de Transplante Cardíaco, Hospital da Unimed João Pessoa, João Pessoa, Paraíba, Brazil; 5 Heart Transplant Program, Hospital de Coração de Messejana, Fortaleza, Ceará, Brazil; 6 Department of Anesthesiology, Pain, and Critical Care Medicine, Universidade Federal de São Paulo (UNIFESP), São Paulo, São Paulo, Brazil; 7 Department of Hospital, Home, and Urgent Care (DAHU), Secretaria de Atenção Especializada à Saúde (SAES), Ministério da Saúde (MS), Brasília, Distrito Federal, Brazil

**Keywords:** Heart Transplantation, Cardiac Surgery, Enhanced Recovery after Cardiac Surgery, Perioperative Care Optimization

## Abstract

Modern healthcare increasingly relies on predictive, proactive, participatory, and personalized paradigms. While Enhanced Recovery After Cardiac Surgery (ERACS) has transformed standard cardiac procedures, its application to heart transplantation (HT) remains unmapped. HT patients exhibit unique vulnerability across physical, psychocognitive, social, and nutritional dimensions. Integrating ERACS pathways into HT protocols represents a critical opportunity to optimize patient-centered recovery. This manuscript addresses this gap by establishing the conceptual link between these fields and proposing a novel framework for their integration.

## INTRODUCTION

**Table:** 

Abbreviations, Acronyms & Symbols	
ERACS	= Enhanced Recovery After Cardiac Surgery
ERACT	= Enhanced Recovery After Cardiac Transplantation
ERAS	= Enhanced Recovery After Surgery
HT	= Heart transplantation
INTERMACS	= Interagency Registry for Mechanically Assisted Circulatory Support
LOS	= Length of stay
POCUS	= Point-of-care ultrasound

In the evolving landscape of cardiac surgery, enhanced recovery protocols are transforming how patients recover, making safety, efficiency, and long-term outcomes their highest priorities^[1]^. Brazil was one of the global epicenters of the early translation of Enhanced Recovery After Surgery (ERAS) concepts into the complexity of cardiac surgery^[2,3]^, the same country manages one of the world’s largest public heart transplantation (HT) programs. In 2023, 424 HTs were performed; mean length of stay (LOS) was 16 days, and mortality was 16.5%. Among 572 wait-listed patients, 27.5% were transplanted within 30 days, and waiting-list mortality reached 24.3%^[4]^. With several centers implementing ERAS across the country, experts are joining forces for a greater challenge. This HT manual^[5]^ provides recommendations for implementing an efficient, safe, and smart recovery protocol for HT by a practical/opinion-based proposal grounded in the literature and the authors’ experience.

## COMMENTS

ERAS represents a paradigm shift in cardiac surgery, transitioning care from isolated interventions to a structured, multidisciplinary methodology which covers the entire perioperative period.

The present review was based on publications available on the Enhanced Recovery After Cardiac Surgery (ERACS) platform (https://www.erascardiac.org/education/publications/) and was complemented by the authors’ practical experience as cardiac/transplant surgery multidisciplinary professionals and with experience in implementing ERACS care pathways in our local setting. Integrating ERACS^[6]^ into HT is essential but challenging mainly due to patient’s multidimensional frailty^[7]^, considering that the waiting list period creates a window of opportunity for prehabilitation, multifaceted patient screening/risk assessment improvement, and patient engagement^[8]^.

It is recommended that a designed “champion” leads a multidisciplinary team (level of evidence in ERACS programs: moderate)^[8]^ to create a locally adapted, evidence-based Enhanced Recovery After Cardiac Transplantation (ERACT) manual (Figure 1) and ensure continuous data collection and element’s adherence audits with continuously updates to the manual and staff training^[6]^ team (level of evidence in ERACS programs: moderate)^[8]^.

**Fig. 1:**
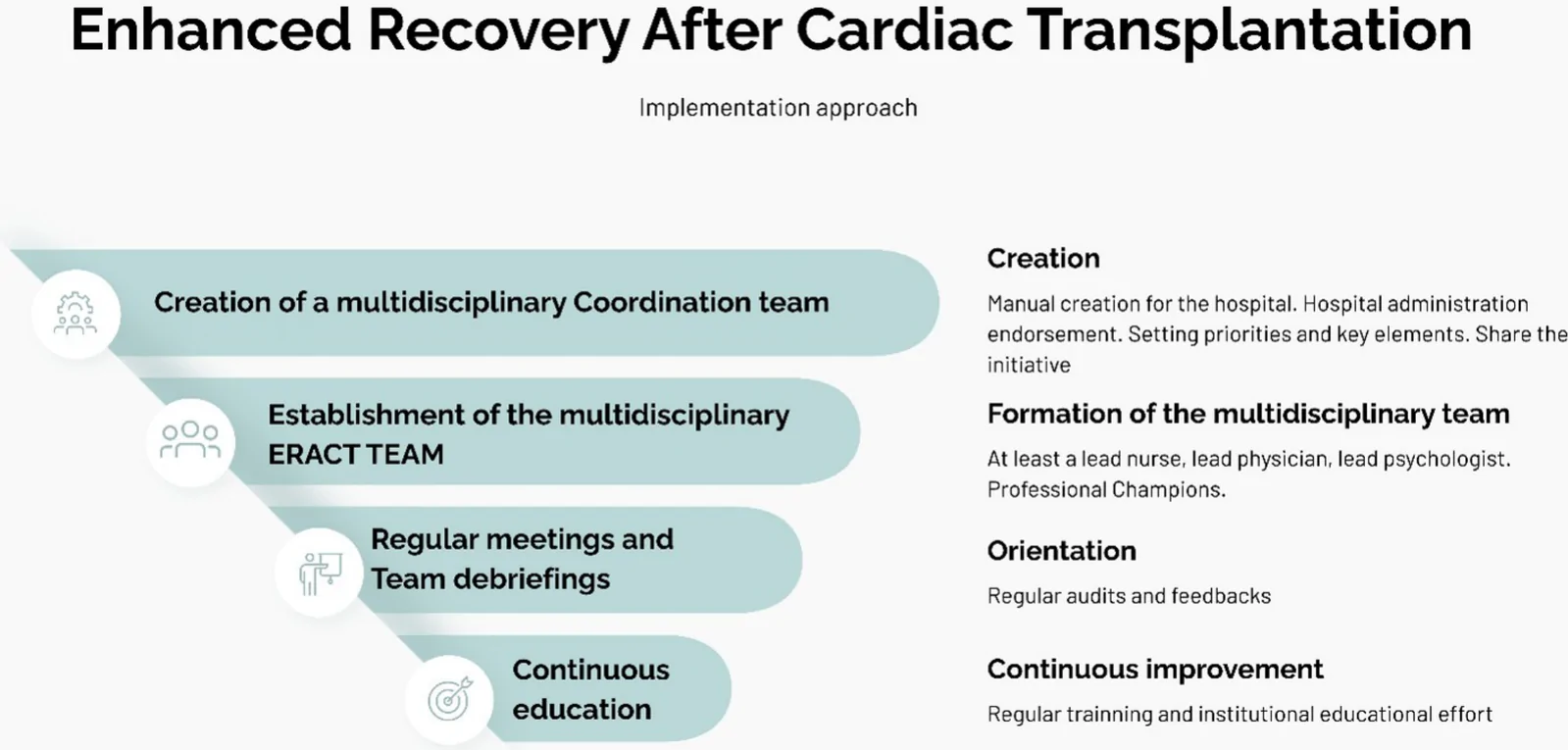
Implementation strategy. ERACT=Enhanced Recovery After Cardiac Transplantation.

ERACT should begin in a lower-risk subgroup (Figure 2) and expand with the learning curve^[6,9]^, given that every journey can be improved through planning and perioperative optimization.

**Fig. 2:**
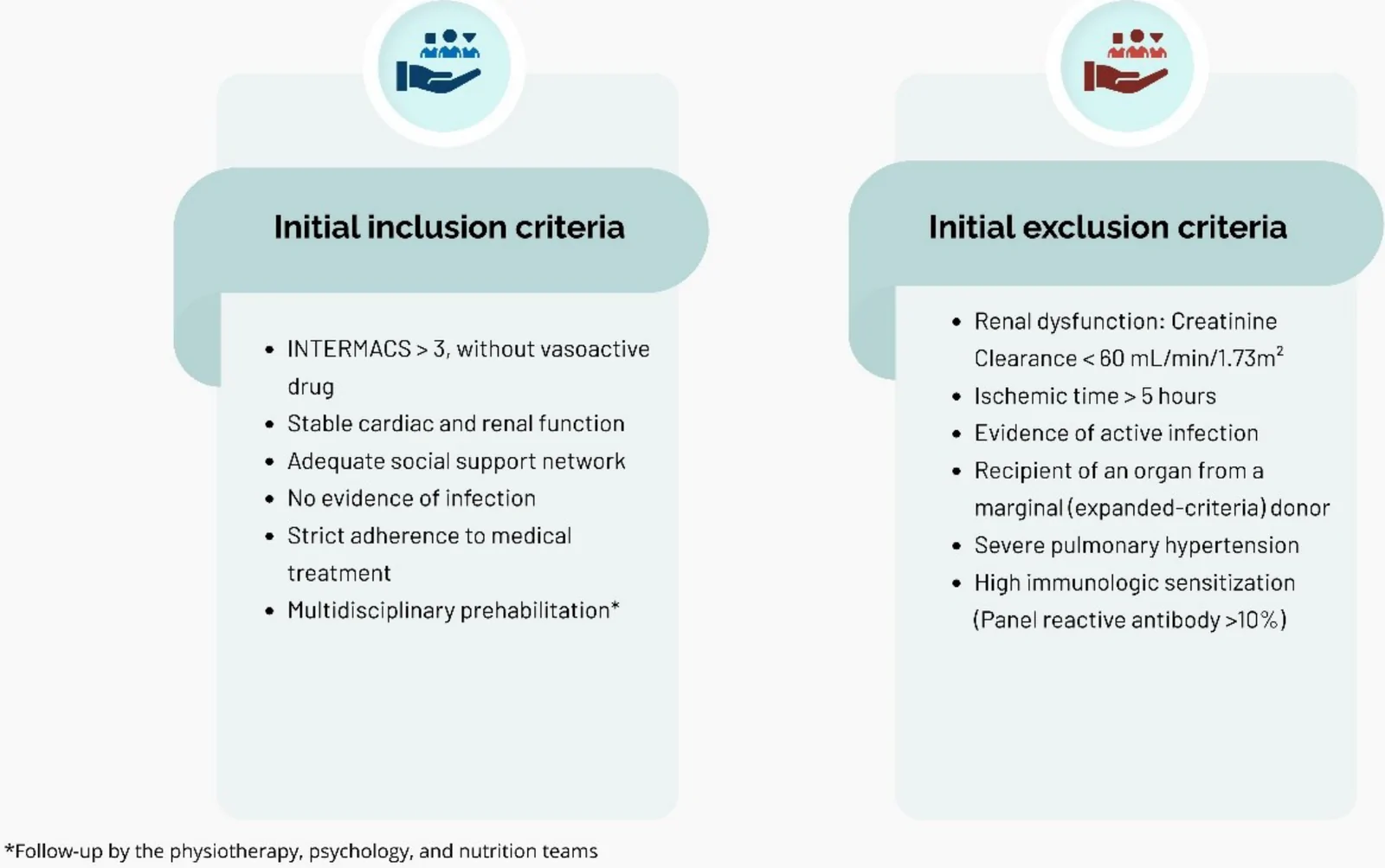
Criteria suggestions. INTERMACS=Interagency Registry for Mechanically Assisted Circulatory Support.

Interventions combine ERACS principles^[8]^, transplant-specific adaptations^[10^–^12]^, and authors expertise, by a practical/opinion-based proposal grounded in the literature and the authors’ experience.

In addition to the impact of education and pre-habilitation, there is evidence that integrates into this journey, such as cardiopulmonary rehabilitation^[13,14]^, nutrition optimization^[15,16]^, proactive pain control^[17]^, and psychological support^[18]^, as well as the rigid closure of the sternum^[19]^ and digital drainage solution^[20]^ to facilitate safe mobilization^[8,21]^, the main focus of ERAS programs. Based on ERACS evidence^[2,9]^ and local context, a seven-day postoperative LOS (target of three LOS in intensive care unit days and four in ward phase) within the ERACT pathway is feasible for most patients with eligible criteria with optimized preoperative phase care, high adherence, and ongoing recovery reinforcement (Figure 3). Discharge should be criteria-based after clinical stability and home readiness, supported with structured hospital follow-up.

**Fig. 3:**
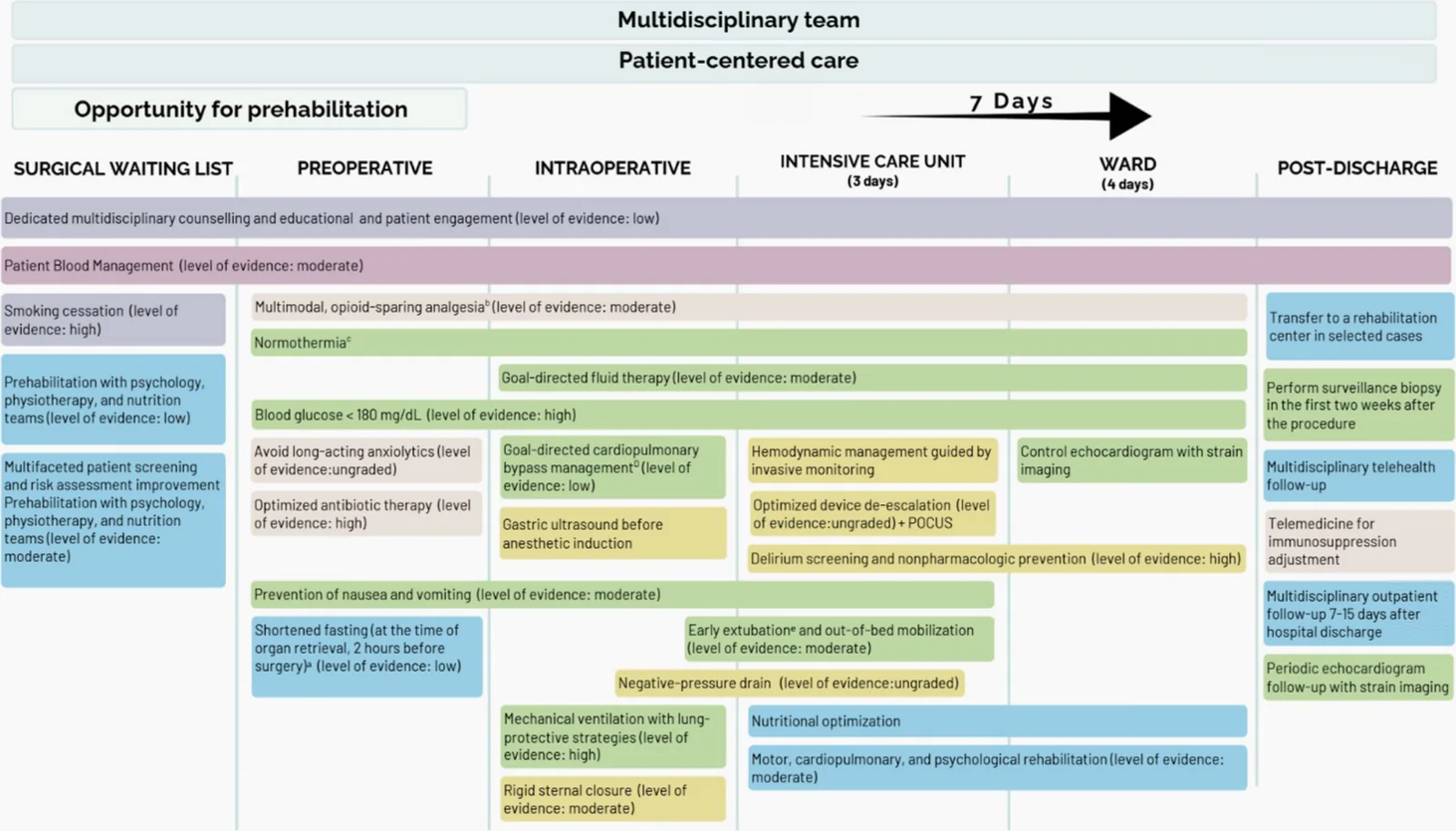
Enhanced Recovery After Cardiac Transplantation’s elements. ^a^Caution in patients at risk of delayed gastric emptying (previous history, gastroparesis, gastroesophageal reflux disease, or use of glucagon-like peptide-1 analogues); ^b^use of regional blocks; ^c^start anastomoses on the left side to allow faster rewarming; ^d^individualize oxygen delivery (DO_2_) and consumption (VO_2_) with continuous or point-of-care monitoring; ^e^≤ 6 hours after admission to the intensive care unit. Level of evidence in Enhanced Recovery After Cardiac Surgery program based on specialty society guidelines^[8,22]^. POCUS=point-of-care ultrasound.

With hospitals under pressure to shorten LOS and prevent readmissions, ERAS offers a roadmap to smart recovery. Evidence indicates that ERACS bundle of interventions are associated with fewer postoperative complications, lower healthcare resource use, and improved patient experience^[2,23,24]^. These gains not only support value-based healthcare but highlight the need to advance perioperative care in HT, promoting continuous improvement and sustainability within the public healthcare system.

The adaptation of ERACS principles to HT represents a promising strategy to improve perioperative care and clinical outcomes of this fragile population, but the adoption requires adaptations tailored to this population, with prehabilitation during the waiting-list emerging as a key opportunity to avoid clinical stagnation and optimize the further steps in recovery. We believe the time has come to move from conceptual discussion to a multicenter pilot implementation, engaging transplant centers in a shared effort to refine, validate, and operationalize this proposed model for ERACT pathway.
